# On a New Method of Performing Iridectomy

**Published:** 1864-01

**Authors:** Julius Homberger


### MISCELLANEOUS.

### ON A NEW METHOD OF PERFORMING IRIDECTOMY.

BY JULIUS HOMBERGER, M. D.,
Editor of the American Journal of Ophthalmology.

A great difficulty in performing iridectomy for the purpose of diminishing intra-ocular pressure, consists in the removal of the iris *to its ciliary insertion.* Another necessity, which is also not easily accomplished in many cases, is the excision *of a large piece* of the iris. As it is necessary to go far beyond the margin of a dilated pupil with a lanceolar knife, in order to get a large corneal wound, the danger arises of injuring the lens, which is considerably pressed forward in glaucoma. Again, the instances are not rare where even experienced assistants fail to cut off the iris to the edge, and thus cause a negative result of the operation.

It is not my intention to analyse or to criticise the different modifications which have been invented by Von Graefe, Arlt, Frobelius, Bowman, and others, with a view to do away with these difficulties. No practical eye-surgeon will deny that, in spite of all modern propositions, the execution of iridectotomy is still attended by the above named inconveniences.—Therefore, though the method which I am going to describe has not yet stood the test of numerous experiments on living subjects, I do not hesitate to recommend it to the readers of this Journal for further trial, confiding in the easiness of its performance and the certain results which it seems to promise.

With a cataract knife, the point of which, directed towards the centre of he globe, is pushed into the sclerotic at a distance of half a line from the margin of the cornea, a linear opening is made, which, by mere pushing forwards of the knife, is lengthened in a radial direction, until the cut reaches three-quarters of a line beyond the edge of the cornea. During the performance of this cut the back of the knife does not for one momens leave its direction towards the centre of the eyeball. The knife is then gradually withdrawn, so that the aqueous humor is slowly evacuated. By this first act of the operation the anterior chamber is opened, and the iris fissured, from its ciliary insertion, up to a point about half a line distant from its periphery.

The second act of the operation consists in the introduction into the wound of one branch of a fine, but strong pair of scissors, slightly curved laterally. The point of one branch of the scissors is introduced along the posterior surface of the cornea into the anterior chamber, and its cutting edge laid into the angle formed by the junction of the iris and cornea.—By one or two movements of the scissors, a wound is produced corresponding with the size of the piece of the iris which is intended to be removed. It will be necessary, in order to introduce the scissors far enough, to enter first but a little way into the wound made by the knife, and to enlarge it by a small, almost rectangular incision.

In the third act, a common iris-forceps is introduced into the anterior chamber, but not in a diagonal direction, as usual. With its points the operator takes hold of that part of the iris next to the angle of the wound, and, by a slight traction (in the direction of a tangent touching the margin of the cornea in the wound,) he tears the already fissured iris up to the pupillar margin, and then, by continued pulling, he severs it from its ciliary insertion. As soon as the iris is torn off up to the opposite angle of the corneal wound, the operator himself, or an assistant, removes the separated segment of the iris, with either knife or scissors.

The advantages of this method I wish to condense in the following points, and would be glad if by my proposition of a more convenient way of performing iridectomy I had contributed a mite to the universal diffusion of this important operation.

1.—The opening in the anterior chamber is made in such a way that the instruments do not in any way come in contact with the pupillary region, and there is therefore no danger of injuring the lens.

2.—The inner edge of the corneal wound is made with much more certainty in the junction of iris and cornea than with either knife or lance.

3.—The tearing of the iris from its insertion loses by the previously made fissure of that membrane the danger of an accidental dialysis, while it insures a peripheral pupil with more certainty than if the iris is cut off after having been dragged out in the manner hitherto practiced.

4.—The cutting off of the iris may be performed by assistants of little experience, because, even if not well executed, it does not, as in the usual methods, make it dangerous or even impossible to resume hold of the iris.

Finally, I may be permitted to remark that I do not consider the division of some fibres of the ciliary muscle (Hancock) of great therapeutical importance, but that I think, that the angular opening, which allows a part, at least, of the aqueous humor to escape for some time, is very favorable to a gradual diminution of intra-ocular pressure. The importance of a compressive bandage during the after-treatment, may, by this circumstance, be considerably lessened, or even totally annulled.—*New York Medical Times.*

